# German general practitioners’ self-reported management of patients with chronic depression

**DOI:** 10.1186/s12888-017-1564-z

**Published:** 2017-12-13

**Authors:** Florian Wolf, Antje Freytag, Sven Schulz, Thomas Lehmann, Susann Schaffer, Horst Christian Vollmar, Thomas Kühlein, Jochen Gensichen

**Affiliations:** 1 Institute of General Practice and Family Medicine, Jena University Hospital, Bachstraße 18, D-07743 Jena, Germany; 2 Institute of Medical Statistics, Computer Sciences and Documentation, Jena University Hospital, Bachstraße 18, D-07743 Jena, Germany; 3 Institute of General Practice, University of Erlangen-Nuremberg, Universitätsstraße 29, D-91054 Erlangen, Germany; 4 Institute of General Practice and Family Medicine, University Hospital of LMU Munich, Pettenkoferstr. 8a/10, D-80336 Munich, Germany

**Keywords:** Chronic depression, Comorbidity, General practice, Quality of health care, Surveys and questionnaires

## Abstract

**Background:**

Patients with chronic depression (persisting symptoms for ≥2 years) are a clinically relevant group with extensive (co)morbidity, high functional impairment and associated costs in primary care. The General Practitioner (GP) is the main health professional attending to these patients. The aim of this study was to examine the GPs’ perception on managing patients with chronic depression.

**Methods:**

We performed an explorative cross-sectional study with a systematic sample of GPs in central Germany. Source of data was a written questionnaire (46 items). Descriptive analysis was carried out.

**Results:**

Two hundred twenty (out of 1000; 22%) GPs participated. 93% of the GPs distinguish between care for patients with chronic depression and acute depressive episode. 92% would recommend psychotherapeutic co-treatment to the chronically depressed patient. 52% of GPs would favour a general restraint on antidepressants (ADs) in older chronically depressed patients (≥ 75 years) whereas 40% suggest long-term pharmacotherapy. If severe physical comorbidity is present GPs would be restrictive in prescribing ADs (65%) or would urgently refer to specialist psychiatric services (40%). In case of a comorbid anxiety disorder 66% of the GPs would suggest a combined psycho- und pharmacotherapy. If a substance use disorder coexists 84% would prefer urgent referrals to specialist services.

**Conclusions:**

Participating GPs report awareness towards chronic depression in their patients. Physical and mental comorbidity seem to play an important role in GPs’ treatment decisions.

## Background

In primary care 10–30% of patients with major depressive disorder develop a chronic course with persistent symptoms of at least 2 years [1, 2]. Patients with chronic depression show higher rates of physical and mental comorbidity as well as more severe and longer lasting functional and psychosocial impairment than individuals with acute episodic depression [3–5]. Consequently, GPs, who are the main health professionals attending to these patients, face severe challenges [6, 7]. There is evidence that due to the specific characteristics, needs and treatment options for this patient group the clinical differentiation between chronic and acute depressive episode is both meaningful and useful [2, 3, 5, 8–10].

Guideline recommendations are predominantly directed to acute forms of depression due to the unsatisfying evidence base for the treatment of chronic courses [11]. Pharmacological and psychotherapeutic treatment of chronically depressed patients is recommended by several clinical practice guidelines including the American Psychiatric Association (APA), the National Institute for Health and Clinical Excellence (NICE) and the German National Disease Management Guidelines (NDMG). Regardless of chronicity antidepressant continuation for at least 24 weeks (APA and NDMG: 4–9 months) is suggested after a successful acute phase [11–13]. Frequent consultations and constant monitoring (evaluation of response, treatment efficacy and tolerability, measures of blood count and liver enzymes, ECG controls) are further required throughout the process of AD treatment [11]. Risk factors supporting a long term (2 years to lifetime) AD maintenance therapy are amongst others older age, chronic episodes and significant mental or physical comorbidity [14]. NICE suggests a maintenance treatment in elderly patients with multiple depressive episodes in order to prevent relapse [13]. Since there are only a few studies performed in primary care settings with follow-up periods longer than 12 weeks and satisfactory sample sizes, appropriate analysis of long-term AD and/or psychotherapeutic effects is currently not possible [15–19].

Evidence for treatment of chronically depressed patients with comorbidity is still limited [20]. Patient-related factors such as old age, severe physical or mental comorbidity are common in primary care patients and may have impact on GPs’ therapeutic decision-making and outcome [21–24]. However, guideline recommendations are mainly based on studies with highly selected patients not complicated by mental and physical comorbidities or social difficulties [25].

Since most treatment recommendations for chronic depression derive from patients in specialist care research is needed to analyse the health care delivery for chronic depression in primary care settings [2, 5].

## Objectives

Main objective: To examine if German GPs distinguish between patients with chronic and acute episodic depression.

Further objectives: To describe the GPs’ self-reported management of patients with chronic depression and to investigate the association between age, physical or mental comorbidity and GPs’ treatment decisions.

## Methods

An explorative cross sectional survey was performed in central Germany from June to August 2015. Expecting a response rate of at least 20% to 30% as obtained in our prior surveys we contacted 1000 German GPs, who were randomly chosen from a database provided by the Association of Statutory Health Insurance Physicians in the federal states of Thuringia and Bavaria. In each case 500 GPs were contacted representing 30% of all Thuringian and 5.5% of all Bavarian GPs. They were asked to complete a written questionnaire anonymously.

The questionnaire was designed and piloted (*n* = 5 GPs) consisting of 46 items. Ten items referred to GPs’ characteristics and 36 items dealt with their management of chronically depressed patients:

- two items covered diagnostic measures (use of validated instruments, screening for comorbidity) [11]
- eleven items dealt with pharmacotherapy (initiation, duration, selection of active agent, switching) [11–14, 20]
- two items approached the therapeutic differences between chronic and acute episodic depression [5, 9, 10, 26]
- seven items asked for the use of additional non-medical approaches (e.g. psychoeducation, frequency of consultations, monitoring, adherence enhancement, crisis management) [11]
- three items concerned the co-treatment by specialists (motives for referral, waiting times, availability) [11, 27]
- seven items addressed patient related-factors relevant for clinical decision making (e.g. advanced age, severe physical or mental comorbidity) [11, 13, 14, 28]
- four items were constructed to estimate the influence of other factors (work incapacity, guideline recommendations, clinical experience, billing and reimbursement) [29, 30]

Since the German National Disease Management Guideline (NDMG) for unipolar depression contains hardly any recommendations for the management of chronic depression, our questionnaire was not aimed to test GPs’ guideline concordance. Nevertheless many of the constructed items were based upon NDMG recommendations for unipolar depression in order to provide a vast spectrum of procedures which have been clinically proven and are based on the best evidence available [11]. A brief case report (68 words) of a chronically depressed patient was introduced in order to homogenate the understanding of the subsequent questions to GPs on their management of chronically depressed patients. Based on prevailing scientific doctrine we provided a definition of chronic course in terms of persisting symptoms for ≥2 years [2, 5, 8, 11]. The central question was if GPs differentiate between chronic and acute episodic depression. Most items including the main question were formulated as closed statements, which could be agreed or disagreed, others were constructed as ratings and participants had to estimate the specific degree of relevance. Answering the questionnaire took about 15 min based on the pilot test. Logical missings were defined in advance.

### Data analysis

Data were transcribed electronically into a database. We performed a descriptive analysis of quantitative variables using SPSS Statistics. Chi-square test was used to examine GPs’ differentiation between chronic and acute depressive episode. Other characteristics were displayed through absolute and relative frequencies. Explorative subgroup analyses (paired comparison) were conducted based on participating GPs’ characteristics (see Table 1). Incomplete data was addressed by performing imputation variance estimation with a total of 20 imputations using the multiple imputation method Markov chain Monte Carlo (MCMC). Moreover, a non-responder analysis was performed by statistical comparison of participating GPs’ and average German GPs’ characteristics.

**Table 1:** Participating GPs’ characteristics

	Total
N	220
Age in years, mean (SD)	54.4 (8.5)
Female, n (%)	120 (54.5)*
Clinical experience in years, mean (SD)	27.2 (9.3)
Qualification of psychosomatic basic medical care, n (%)**	178 (80.9)
Location of GP’s practice: rather rural, n (%)	111 (50.5)
Profile of GP’s practice: single, n (%)	130 (59.1)*
Patients per quarter, mean (SD)	1048.4 (351.1)*
Number of patients per quarter treated for depression, mean (SD)	104.8 (103.8)
Number of patients per quarter treated for chronic depression, mean (SD)	60.0 (62.8)

*significant difference (*p* < .05) compared to overall average of German GPs (due to specific characteristics of GPs from Thuringia)

**Additional qualification needed for monetary compensation of psychosocial exploration, counseling, educational and motivational techniques. Established in the 1990s the curriculum aims to improve quality of care and has become mandatory for all vocational trainees who want to specialize in the discipline of General Practice

The study protocol was approved by the local ethics committee (reference number: 4454–06/15) and we report the results in the line with the STROBE standards [31].

## Results

220 GPs (22% of the 1000 contacted GPs) participated in our survey. They were 54 years old on average, more than half female, and they treated about 105 patients with depression per quarter of a year (mean), 60% of these with chronic depression (see Table 1).

Extensive missing data was recorded in only one participant who had to be excluded from the analysis. Over all items of the questionnaire, the widest between imputation variance observed was 3.7% (range from −1.9 to +1.8).

93% of the participating GPs stated that they distinguish patients with chronic depression from patients with acute depressive episode (chi-square test; *p* < 0.01). They reported to start AD treatment earlier (44%) and to prescribe for longer time (69%) as well as to intensify monitoring and follow-up (61%). 47% of GPs reported a rather early referral to mental health care specialists.

Half of the GPs reported to use validated instruments for diagnostic and/or monitoring purposes regularly. 92% would recommend psychotherapeutic co-treatment to their chronically depressed patients. About 84% of primary care patients with chronic depression are prescribed ADs. If initiated, a guideline-concordant length of AD prescription (≥ 24 weeks) would be applied in approximately 60% of the cases. Over 92% of GPs indicated to provide weekly consultations during acute phase of two months. Monitoring of blood count and liver enzymes (96%) as well as routine ECGs (89%) were stated to be part and parcel of GPs’ care. 80% of GPs would utilize switching of ADs in case of non-response without a prior consultation of a specialist. 56% declared to screen for psychiatric comorbidities, 56% reported that they use psychoeducational methods, 59% adherence enhancement strategies and 50% develop crisis management plans when dealing with chronically depressed patients.

About 80% of participating GPs declared patient’s age (75 years and older) as a relevant factor of clinical decision making: 90% rated severe physical comorbidity as relevant, 97% comorbid anxiety disorder and 95% comorbid substance use disorder.

Most GPs favour a restraint on medication (52%) in older patients with chronic depression but on the other hand nearly 40% argue for long-term pharmacotherapy (see Table 2). The presence of severe physical comorbidity in patients with chronic depression leads GPs to either holding back on ADs (65%) or urgent referrals to mental health care specialists (40%). Long-term medication (13%), the necessity of psychotherapy (15%) or combination therapy (20%) seem to be less important to participating GPs. 66% of GPs see the need for combination therapy when comorbid anxiety disorder is coexistent with chronic depression. 32% feel that psychotherapy is essential. About 40% prefer an urgent referral to the specialist. The factor comorbid substance abuse mainly (84%) goes along with GP’s urgent referral. Only 12% of GPs believe that an urgent referral to a mental health care specialist is needed when their chronically depressed patient is of high age. Psychotherapy alone (3%) or a combination of pharmacotherapy and psychotherapy (14%) play a subordinate role in GPs’ perceived management of chronically depressed patients of advanced age.

**Table 2:** Therapeutic consequences of patient-related factors present in primary care patients with chronic depression (in percent)

patient related-factor	relevant for clinical decision making (yes)	Therapeutic consequences, when the patient-related factor is perceived to be relevant (multiple selections possible)				
		Restriction in medication	Long-term pharmaco-therapy	Need for psycho-therapy	Need for combination therapy	Urgent referral to specialist
advanced age	80.2	51.7	39.7	3.4	14.4	12.1
severe physical comorbidity	90.7	65.1	12.5	15.1	20.3	39.6
cormorbid anxiety disorder	92.7	1.0	21.7	32.3	66.2	40.4
comorbid substance abuse	95.4	29.1	8.7	19.4	24.8	83.5

No significant differences regarding the reported results (main and additional questions) could be obtained when GPs were paired by age, gender, years of clinical experience, practice location or by the number of patients treated per quarter. When stratified by practice profile, GPs working in a practice cooperation reported to carry out weekly consultations (Chi-square: 4.55; double-sided significance: .033) and ECG-controls (Chi-square: 9.28; double-sided significance: .002) more frequently than their colleagues working single-handed. GPs with the additional qualification of psychosomatic basic medical care stated to perform monitoring of blood count and liver enzymes (Chi-square: 5.29; double-sided significance: .021) as well as routine ECGs (Chi-square: 5.73; double-sided significance: .017) more often that GPs without this qualification.

## Discussion

We studied German GPs’ self-reported management of patients with chronic depression. The vast majority of the GPs differentiated between chronic and acute episodic depression as recommended by several authors [2, 3, 5, 9, 10, 26].

The 220 participating GPs consider chronic depression as amenable to treatment and GPs’ reported interventions are extensive. These findings correspond with the implications of a systematic review from Michalak and Lam [32] and a mixed-method approach by Fleury et al. [30]. ECG controls and monitoring of blood count and liver enzymes are generally performed by participating GPs. This goes along with guideline recommendations [11]. ADs are prescribed to 84% of the chronically depressed patients. Vuorilehto et al. [33] report that 82% of patients with major depressive disorder (MDD) or partly remitted MDD are prescribed ADs but only half started and adequately adhered to AD treatment. Participating GPs feel confident in switching ADs in case of non-response without prior consultation of a specialist. This seems remarkable, since it is not in the line with guideline recommendations [11, 34]. In our study the majority of chronically depressed primary care patients would receive an AD medication ≥24 weeks [11–13]. It is considerably higher (60%) compared to claims data analyses (40%) [35, 36]. This contrast may be explained by a tendency of claims data analyses to overestimate the number of patients with chronic depression as a consequence of a perpetuated administrative documentation of diagnosis codes. Due to the small number of studies investigating the effectiveness of psychotherapeutic treatment among chronically depressed primary care patients it remains unclear if the GP-reported perceptions of the suitability of psychotherapeutic co-treatment (92%) are based on solid evidence. The reported numerous consultations, the frequent use of validated instruments for diagnostic and/or monitoring purposes as well as the reported effort to screen for psychiatric comorbidity are in contrast to everyday experience [37] and might result from desirability and willingness rather than actual implementation. Half of the participating GPs reported to use psychoeducational methods, adherence enhancement strategies and crisis management plans. This finding is supported by rising evidence for such “low intensity psychosocial interventions” but so far the effectiveness related to chronically depressed primary care patients has not been systematically investigated [11, 13, 38]. Nevertheless, relating to low intensity psychosocial interventions NDMG highlights the coordinative role that GPs can play in the context of multimorbidity and in order to prevent further fragmentation of care [11]. Though, it has to be considered that a simple guideline implementation strategy might be ineffective for improving the management of depression in primary care settings [39–41]. A tailored intervention (including outreach visits, web-based recommendations and recourses, management tools) to implement guideline recommendations for elderly patients with depression in primary care did in fact raise guideline adherence of participating GPs but did not show relevant differences between the intervention and control groups in terms of depression severity, anxiety, loneliness, contact with voluntary organisations, physical activity, utilisation of self-help programmes and medication adherence [42]. It is argued that depression guideline recommendations can be made more relevant and applicable for primary care if they addressed the effectiveness of different treatment options and focused on the integration of relevant social factors, comorbidities as well as the patients’ individual needs and preferences [25, 28, 43, 44].

The reported treatment of chronically depressed patients aged 75 years or more is heterogeneous. There seem to be two lines of GPs managing the chronically depressed elderly: Going along with the findings of Linden and Kurtz [24], one line (about 50%) states that older age implies a restraint in AD medication although guideline recommendations have highlighted that ADs are also effective in elderly patients with a stronger focus on side effects and compatibility [11, 13]. The other line (about 40%) holds that a systematic long-term medication is especially important in patients of advanced age going along with NICE recommendations on elderly patients with multiple depressive episodes [13]. There is only a small fraction of GPs (12%) believing that an urgent referral to a mental health care specialist is needed when their chronically depressed patient is of high age. More generally expressed, psychotherapy seems to play a subordinate role in GPs’ management of chronically depressed patients of older age [24]. These findings go along with results from claims data analyses presuming that advanced age is a predictor for withholding psychotherapeutic interventions [36]. Clinically, the low rates may result from difficulties for the provision of psychotherapy to older patients because of limited mobility, somatic comorbidity or sensory disabilities [11].

The presence of a severe physical comorbidity (e.g. heart failure, cancer, COPD) seems to have a strong effect (91%) on GPs’ treatment decisions in terms of an urgent referral to a psychiatric specialist. Concerning the reported restraint in ADs in this patient group (52%) our findings are in line with Coventry et al. [29]. Nevertheless, there are contradictory findings that chronic physical comorbidity does not consistently lead to lower quality of depression treatment or follow-up care in depressed primary care patients [29, 45].

Comorbid mental disorders are valued as highly relevant. Substance use disorder is faced with urgent referrals (84%) whereas anxiety disorder is mainly (66%) dealt with by combined pharmacological and psychotherapeutic treatment.

Piek et al. found that chronicity, suicidal tendency, the perceived need for psychotherapy and the number of ADs used induced GPs to refer depressive patients to mental health care specialists [27]. However, the authors proposed that other factors must play a part in GPs’ decision for referral since only 8–11% of variance could be explained. Our findings suggest that the presence of patient-related factors such as severe physical comorbidity and mental comorbidity might determine whether GPs refer chronically depressed patients to mental health care specialists.

Remarkably, GPs’ self-reported management showed high consistency in subgroup analyses. This result suggests that participating GPs’ perceptions and reported therapeutic measures are independent from GPs’ characteristics like their age, gender, clinical experience or location of practice.

### Limitations

The low response rate (22%) was in accordance with the response to postal questionnaire surveys among GPs obtained by Cottrell et al. [46]. From other studies we know that non-responders are of higher age, more experienced, single handed, more stressed (measured by number of patients per quarter) and less well qualified than responding GPs [47]. The comparison of the characteristics of the 220 GPs, who have participated in the survey, with the basic population of German GPs, did not show any deviations in the mentioned variables. The main divergence between participating GPs and the basic population of GPs in Germany is due to the specific distribution of GPs from Thuringia who represent 54% of our sample. GPs from Thuringia are rather female (62% vs. 42%), single handed (69% vs. 57.5%) and treat more patients per quarter (1116.4 vs. 848.7) than average German GPs. However, the mentioned characteristics did not seem to influence GPs’ attitudes and reported management when subgroup analyses were carried out. To sum up, selection bias cannot fully be excluded and may limit the results.

Another limitation of our study is a possible misclassification bias due to a non-validated questionnaire. But between imputation variances showed no substantial or systematic effect due to incomplete data. Since a case report and a definition of chronic course have been provided, GPs’ responses might have been influenced by this information.

We do not know if participating GPs used patients’ records and electronic administrative data while completing the questionnaire or if they simply based their statements on estimation and intuition. Therefore, we can neither exclude social desirability bias nor overestimation respectively underestimation of GPs’ reported actions and perceptions.

## Conclusion

GPs report high awareness towards chronic depression. Advanced age, severe physical and mental comorbidity seem to play an important role in GPs’ treatment decisions in chronic depression care. Possibly, the GPs’ coordinative role in counselling and performing low intensity psychosocial interventions needs further advancement. Our findings may help to improve the management of chronic depression in primary care.

## Abbreviations

AD: antidepressant
APA: American Psychiatric Association
COPD: chronic obstructive pulmonary disease
ECG: electrocardiogram
GP: General Practitioner
MCMC: Markov chain Monte Carlo
MDD: major depressive disorder
NDMG: German National Disease Management Guidelines
NICE: National Institute for Health and Clinical Excellence
SPSS: Statistical Package for the Social Sciences
STROBE: STrengthening the Reporting of OBservational studies in Epidemiology
